# Immunomodulation by cannabidiol in bovine primary ruminal epithelial cells

**DOI:** 10.1186/s12917-023-03756-4

**Published:** 2023-10-16

**Authors:** C. Kent-Dennis, James L. Klotz

**Affiliations:** USDA-ARS Forage-Animal Production Research Unit, University of Kentucky Campus, 1100 S. Limestone Rd. N222J Ag. Science North, Lexington, KY 40546 USA

**Keywords:** Ruminant, Epithelial cells, Cannabinoids, Inflammation, Lipopolysaccharide

## Abstract

**Background:**

Ruminant livestock experience a number of challenges, including high concentrate diets, weaning and transport, which can increase their risk of disorders such as ruminal acidosis, and the associated inflammation of the ruminal epithelium. Cannabidiol (CBD), a phytochemical from hemp (*Cannabis sativa*), is a promising target as a therapy for gastrointestinal inflammation, and may be extremely valuable as either a treatment or prophylactic. However, the effects of CBD in the the ruminant gastrointestinal tract have not been explored, in part due to the restrictions on feeding hemp to livestock. Therefore, the objective of this study was to investigate the immunomodulatory properties of CBD using a model of inflammation in primary ruminal epithelial cells (REC). In addition, CBD dose was evaluated for possible cytotoxic effects.

**Results:**

Negative effects on cell viability were not observed when REC were exposed to 10 μM CBD. However, when the dose was increased to 50 μM for 24 h, there was a significant cytotoxic effect. When 10 μM CBD was added to culture media as treatment for inflammation induced with lipopolysaccharide (LPS), expression of genes encoding for pro-inflammatory cytokine *IL1B* was less compared to LPS exposure alone, and CBD resulted in a down-regulation of *IL6*. As a pre-treatment, prior to LPS exposure, REC had decreased expression of *IL6* and *CXCL10* while CBD was present in the media, but not when it was removed prior to addition of LPS.

**Conclusions:**

Results suggest that CBD may reduce cytokine transcription both during LPS-induced inflammation and when used preventatively, although these effects were dependent on its continued presence in the culture media. Overall, these experiments provide evidence of an immunomodulatory effect by CBD during a pro-inflammatory response in primary REC in culture.

## Introduction

The study of phytochemicals, also called plant secondary metabolites, has gained traction in recent years. Much of this increasing interest is related to their potential bioactive effects, with particular focus on physiological processes related to metabolism and health [1, 2]. In ruminant production, phytochemicals with the capability of altering feed intake, ruminal fermentation, production and disease mitigation are highly sought after, especially with the increasing pressure to find alternatives to antibiotics and traditional means of improving production efficiency [3, 4]. A number of phytochemicals have been shown to have bioactive effects on animal performance. For example, blended essential oils which can be derived from mint, cloves and anise were found to have a positive effect on milk and milk component yield when fed to dairy cows [5]. In another study, Harlow et al. [6] demonstrated that biochanin A, a compound in red clover, may counteract some of the negative effects during a subacute ruminal acidosis challenge.

One class of phytochemicals of particular interest are the cannabinoids, such as cannabidiol (CBD), which are derived from the *Cannabis sativa* plant. Although the benefits and efficacy of CBD are still being determined, there is growing evidence for immune-modulatory effects in humans and rodent models [7]. In fact, CBD as a novel pharmaceutical is the subject of numerous human clinical trials at various stages of completion [8].

Recently, ruminants have been identified as a potential target species for consumption of byproducts of low delta-9-tetrahydrocannabinol (THC) *Cannabis sativa* also known as hemp. This could create a secondary benefit from the large amount of waste biomass produced from processing hemp for cannabinoid extraction or grain harvest [9]. Although the levels of CBD in these byproducts are often relatively low [10], there is evidence that even trace concentrations of cannabinoids can elicit bioactive effects [11]. Currently, hemp and hemp byproducts are prohibited as feedstuffs for livestock entering the human food chain. The status may change, however, pending the completion of research demonstrating its safety with regards to THC contamination, and there may be future opportunities for utilizing these products as low cost feed ingredients with added potential benefits resulting from residual CBD. In addition, as more information becomes available about the effects and efficacy of compounds such as CBD, there may be value in utilizing the isolated compounds directly as a feed supplement.

A promising target for the immune-modulatory effects of CBD is inflammation of the gastrointestinal mucosa [12]. Previous work has suggested that cannabinoids may elicit protection against intestinal inflammation [13]. Cannabidiol had an anti-inflammatory effect in acutely inflamed colonic explants [14]. In ruminants, especially feedlot cattle and high producing dairy cows [15, 16], the use of high concentrate diets to meet energy requirements can result in ruminal acidosis and subsequent complications such as inflammation of the ruminal epithelium [17]. Additionally, several events in the production system, such as weaning and shipping, predispose ruminants to stress and disease and result in economic losses associated with reduced weight gain [18]. A compound such as CBD could be have value as a treatment for disorders such as ruminal acidosis, thereby reducing the cell damage to the ruminal epithelium caused by a prolonged local, inflammatory response [17]. Alternatively, there has been interest in providing “pre-treatments”, such as non-steroidal anti-inflammatory drugs, to cattle prior to stress events, such as transport [19], and cannabidiol may hold prophylactic properties that could be useful for reducing the severity of inflammation during these stress events in ruminants. However, due to the restrictions on feeding hemp or hemp products to livestock, few studies have been conducted to look at the use of CBD or other hemp derivatives in ruminants, and evidence must be extrapolated from experiments with humans or rodents.

Recently, we have demonstrated, with a primary cell culture model, that ruminal epithelial cells (REC) are capable of responding to microbial-derived lipopolysaccharide (LPS) and eliciting a pro-inflammatory response [20]. This model is ideally suited to initially examine the effects of CBD on the immune response in gastrointestinal epithelial cells. Therefore, the aim of this study is to investigate the immunomodulatory effects of CBD exposure on the pro-inflammatory response to LPS in primary REC in culture. More specifically, we have used this model to evaluate the potential benefits of CBD when used as a treatment or prophylactically.

## Materials and methods

### Animals and tissue collection

Tissues used for this experiment were acquired from the abattoir post-slaughter. As live animals were not used in the study, animal care approval was not required. Ruminal epithelial tissue was obtained from Holstein steers (n = 7) that weighed approximately 250 kg and were between 10 and 12 months old. All steers originated from the University of Kentucky beef farm and were fed an alfalfa-based diet prior to slaughter. Steers were slaughtered by captive bolt followed by exsanguination and the tissue was collected within 30 min of death. The ruminal epithelial tissue was excised from the craniodorsal sac and washed vigorously in ice-cold Ca^2+^- and Mg^2+^-free Dulbecco’s phosphate buffered saline (DPBS; Sigma) containing antibiotics with a final concentration of 400 U/mL penicillin, 400 μg/mL streptomycin, 1 μg/mL amphotericin B (Thermo) and 240 U/mL Nystatin (Sigma). The buffer was also used to transport the tissue to the laboratory. Transport and subsequent washing of the tissue occurred within 2 h of collection.

### Primary cell isolation and culture

Isolation and culture of ruminal epithelial cells (REC) are described in detail by [20]. Briefly, ruminal papillae were clipped off at their base and washed thoroughly in DPBS. Serial trypsinization of the ruminal papillae was then performed using a trypsin-EDTA solution (0.25% trypsin and 0.02% ethylenediaminetetraacetic acid; Sigma), with 30-minute incubations at 37ºC. Fractions 3 through 6 were collected, strained through sterile gauze and centrifuged at 200 × *g* at room temperature for 5 min. Cell pellets were then washed three times and re-suspended in M199 cell culture media (Sigma) supplemented with 15% (v/v) fetal bovine serum (Thermo), 20 m*M* N-2-hydroxyethylpiperazine-N’-2-ethanesulfonic acid (HEPES; Sigma), 1X Antibiotic-Antimycotic (Thermo), 240 U/mL nystatin (Sigma), 50 mg/L gentamycin (Sigma) and 100 mg/L kanamycin (Thermo). Cells were seeded into 60-mm culture plates coated with bovine collagen I (Thermo) and placed in a humidified incubator with temperature of 37ºC and 5% CO_2_. On day 1 following isolation, plates were washed with DPBS and the media was replaced. On day 2, plates were washed again and media was switched to minimum essential media (MEM) supplemented with 10% (v/v) fetal bovine serum (Thermo), 20 m*M* HEPES (Sigma), 1X non-essential amino acids (Sigma) and 1X Antibiotic-Antimycotic (Thermo). Cells were grown in plates for approximately 10 days, replacing the MEM every 2–3 days. When plates were > 80% confluent, cells were re-seeded into 24-well plates at a rate of 5 × 10^4^ cells/mL and grown until 90% confluent, at which point they were used for the CBD exposure experiments.

### Preliminary experiment: effect of CBD on REC viability

A subset of REC samples (n = 4) were exposed to 0, 10 or 50 μM cannabidiol (CBD; Cannabidiol solution in methanol, Sigma C-045), or equivalent levels of vehicle only (1:1 methanol:dimethylsulfoxide) in a volume of 0.5 mL/well in a 24-well plate. Cells were exposed for either 6 or 24 h and subsequently evaluated using an alamarBlue (Thermo) viability assay. Following exposure time, alamarBlue dye was added to each well at 1/10th the volume of media. Plates were placed in the incubator for 1 h. Media from each treatment well was then transferred to a flat-bottom 96-well plate in 100 μl duplicates and absorbance was measured at 570 and 600 nm as the reference wavelength. To evaluate viability, color change associated with the cellular reduction of resazurin to resorufin was quantified as the percent difference between treated and control cells [21] using the following equation:

$$\frac{{{\rm{(O2}}\,{\rm{ \times }}\,{\rm{A1)}}\,{\rm{ - }}\,{\rm{(O1}}\,{\rm{ \times }}\,{\rm{A2)}}}}{{{\rm{(O2}}\,{\rm{ \times }}\,{\rm{P1)}}\,{\rm{ - }}\,{\rm{(O1}}\,{\rm{ \times }}\,{\rm{P2)}}}}\,{\rm{ \times }}\,{\rm{100}}$$

Where O1 = molar extinction equivalent of oxidized alamarBlue at 570 nm.

O2 = molar extinction equivalent of oxidized alamarBlue at 600 nm.

A1 = absorbance of treatment wells (CBD or vehicle) at 570 nm.

A2 = absorbance of treatment wells (CBD or vehicle) at 600 nm.

P1 = absorbance of control well (media alone + cells) at 570 nm.

P2 = absorbance of control well (media alone + cells) at 600 nm.

### Experiment 1: can CBD mitigate an established, LPS-induced inflammatory response?

**Fig. 1:**
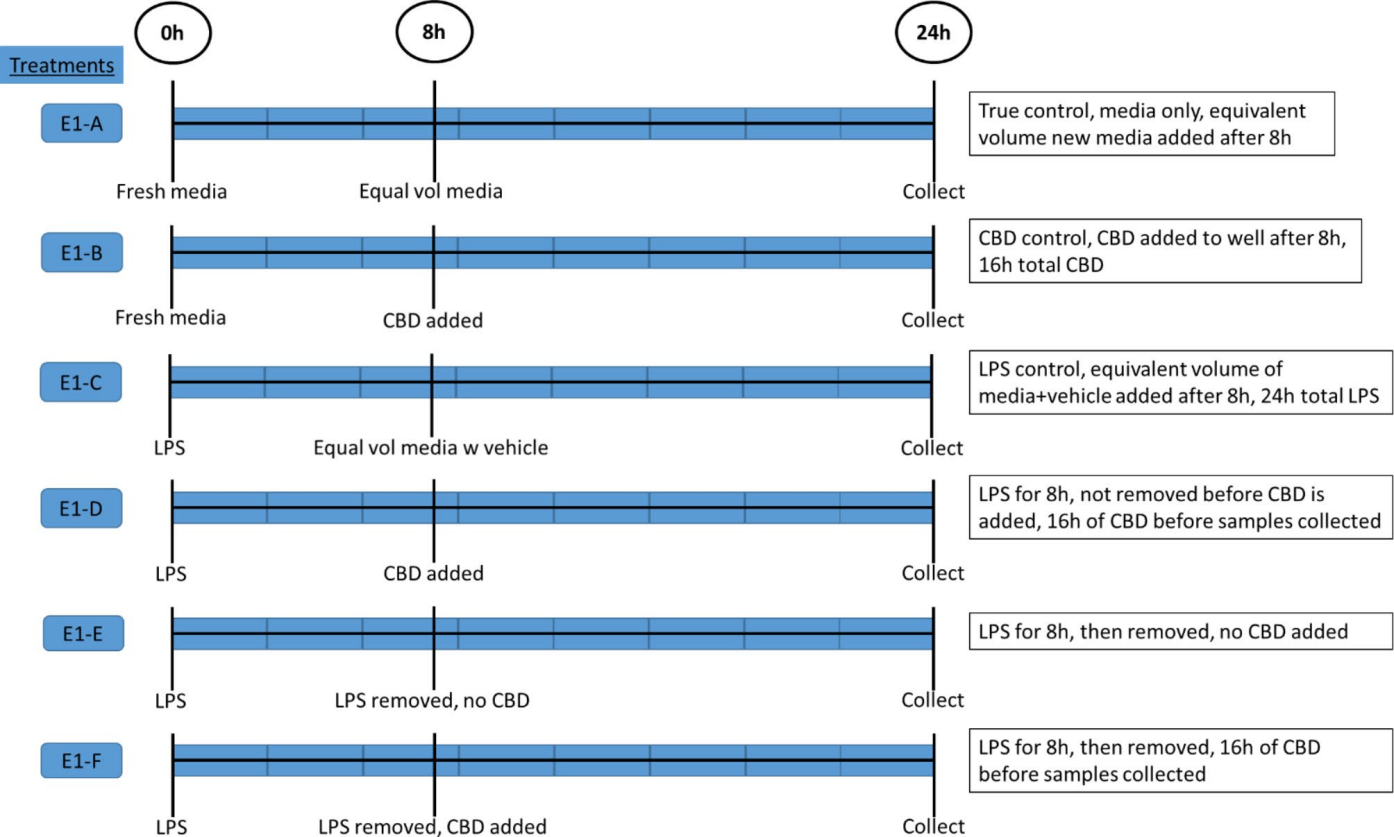
Experimental procedure and treatment descriptions for Experiment 1: Media only control (E1-A); Media added at 0 h, CBD added at 8 h (E1-B); LPS added at 0 h and remained for entire duration (E1-C); LPS added at 0 h, CBD added at 8 h, LPS not removed for 24-h duration (E1-D); LPS added at 0 h then removed at 8 h (E1-E); LPS added at 0 h, then removed at 8 h, CBD added at 8 h (E1-F)

Treatments were administered to duplicate wells of 24-well plates when the REC reached 90% confluence. The final concentration of CBD used was 10 μM, as determined by the preliminary experiment. Lipopolysaccaride is commonly used to induce experimental inflammation. A dose of 1000 EU/mL LPS was added to the cell culture media to induce a pro-inflammatory response. Exposure of REC to LPS results in substantially greater transcript abundance of pro-inflammatory cytokines, as previously demonstrated by Kent-Dennis et al. [20], and therefore provides a repeatable model for investigating inflammation in cell culture. The experimental procedure, exposure times and treatment descriptions are presented in Fig. 1. Six treatments were administered as follows: (1) Media only control (E1-A), (2) Media added at 0 h, CBD added at 8 h (E1-B), (3) LPS added at 0 h and remained for entire duration (E1-C), (4) LPS added at 0 h, CBD added at 8 h, LPS not removed for 24-h duration (E1-D), (5) LPS added at 0 h then removed at 8 h (E1-E) and (6) LPS added at 0 h, then removed at 8 h, CBD added at 8 h (E1-F). When the experiment was complete, after 24 h, the duplicate wells (for each treatment) were lysed and pooled in 1 mL of Trizol (Thermo) and frozen at -80ºC.

### Experiment 2: can CBD prevent or dampen an LPS-induced inflammatory response?

**Fig. 2:**
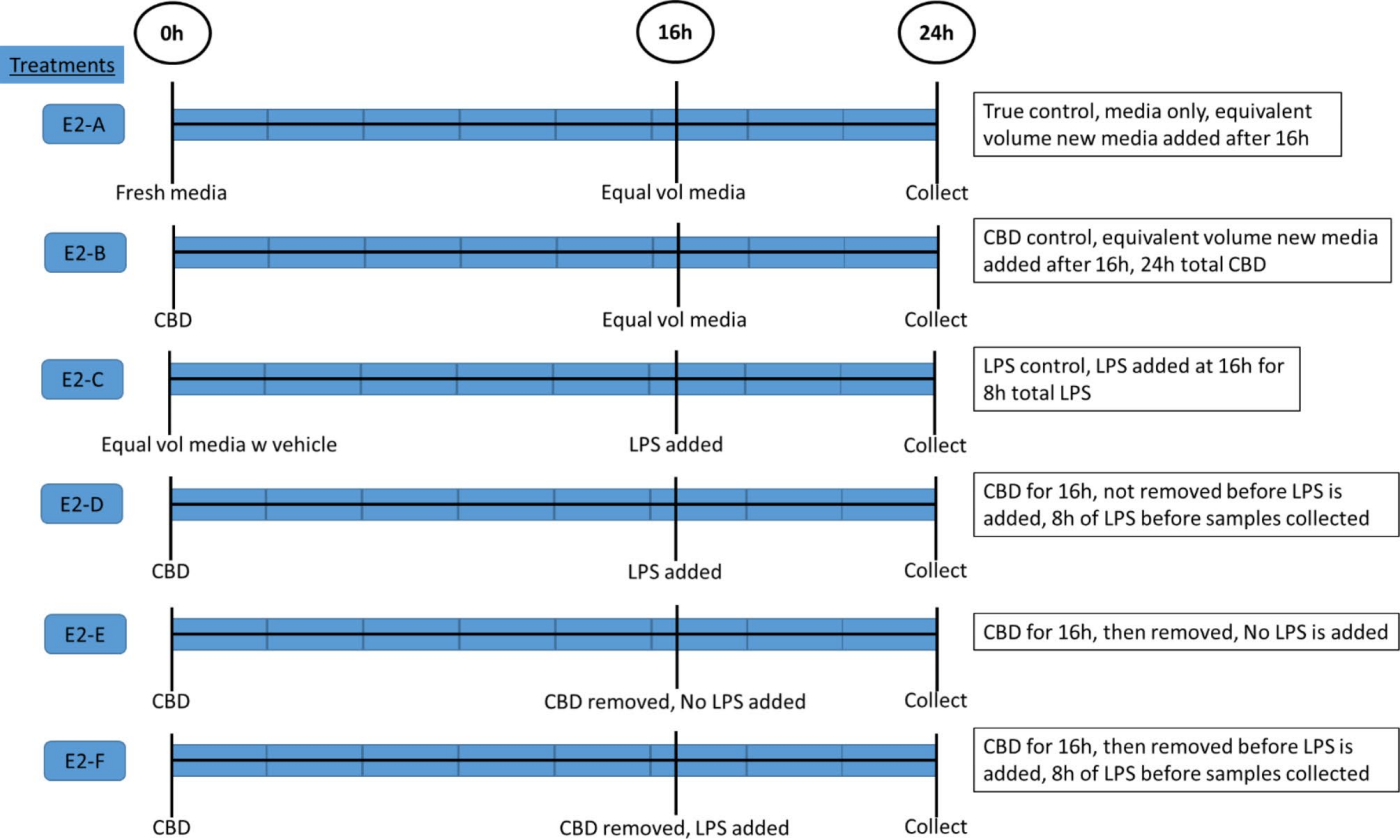
Experimental procedure and treatment descriptions for Experiment 2: Media only control (E2-A); CBD added at 0 h and remained for entire duration (E2-B); media added at 0 h, LPS added at 16 h (E2-C); CBD added at 0 h, LPS added at 16 h, CBD not removed (E2-D); CBD added at 0 h then removed at 16 h (E2-E); CBD added at 0 h, then removed at 16 h, LPS added at 16 h (E2-F)

Treatment administration and sample collected was similar to Experiment 1, with the same CBD and LPS concentrations used. Figure 2 outlines the experimental procedure, exposure times and treatment descriptions. Treatments for Experiment 2 were as follows: (1) Media only control (E2-A), (2) CBD added at 0 h and remained for entire duration (E2-B), (3) media added at 0 h, LPS added at 16 h (E2-C), (4) CBD added at 0 h, LPS added at 16 h, CBD not removed (E2-D), (5) CBD added at 0 h then removed at 16 h (E2-E) and (6) CBD added at 0 h, then removed at 16 h, LPS added at 16 h (E2-F). Again, at the conclusion of experiment, after 24 h, the duplicate wells (for each treatment) were lysed and pooled in 1 mL of Trizol (Thermo) and frozen at -80ºC.

### Total RNA extraction and real time qPCR analysis

Isolation of total RNA was performed using a phenol-chloroform extraction method, with the addition of two isopropanol precipitations as previously described by [22]. Linear acrylamide (Thermo) was used as a co-precipitant in order to visualize the pellet. All samples were DNase-treated using the Turbo DNase kit (Thermo), and RNA integrity was verified using a Bioanalyzer (Agilent Technologies, Santa Clara, CA, USA), and all samples were confirmed to have RNA integrity numbers (RIN) of at least 8. Two micrograms of RNA were reverse transcribed using the High Capacity cDNA reverse transcription kit (Thermo) and the cDNA was diluted to a final concentration of 10 ng/μl with nuclease-free water. Quantitative real-time PCR (qRT-PCR) was performed with 20 ng of cDNA and run in duplicate using Fast SYBR Green Master Mix (Thermo) in a StepOnePlus Real-Time PCR system (Thermo). Where possible, primers were designed to span exon-exon junctions, and subsequently verified for production of a single product with melt curves following amplification. Primers used, including housekeeping genes and target genes of interest, are listed in Table 1. Primers were validated for efficiency via a serial dilution of pooled cDNA, and all primer sets used were between 90 and 112%. Gene expression of target genes was normalized to the geometric mean of two stable housekeeping genes, *GAPDH* and *STX5*. Statistical analysis was performed on ΔCt, while data are presented as fold change, with treatments held relative to the value of the control within each animal.

**Table 1:** Gene-specific primer sequences

Gene ID	Gene Name	Sequence (5’-3’)^1^	Amplicon Size (bp)	Efficiency (%)^2^	Target RefSeq^3^
Housekeeping					
*GAPDH*	Glyceraldehyde-3-phosphate dehydrogenase	F: GGGTCATCATCTCTGCACCT R: GGAGGCATTGCTGACAATCT	101	91	NM_001034034.2
*STX5*	Syntaxin 5	F: CCATTCAGAGGATCGACGAG R: GGATGTGACCGACTGGAAGT	95	103	NM_001075444.1
Targets					
*CASP4*	Caspase 4	F: CACTCGTCTGGCTCTCATCA R: GTCCCTGGCTGTGAGTTTCT	148	97	NM_176638.5
*CXCL8*	C-X-C motif chemokine ligand 8	F: AGAGCTGAGAAGCAAGATCCA R: ACCCTACACCAGACCCACAC	150	104	NM_173925.2
*CXCL9*	C-X-C motif chemokine ligand 9	F: ATTTGCTCCAAGCCCTTCTT R: CTTTTGGTTGACCTGTTTCTCC	136	111	NM_001113172.1
*CXCL10*	C-X-C motif chemokine ligand 10	F: CGAGATTATTGCCACAATGAAA R: CTCTTTCCGTGTTCGAGGAG	130	105	NM_001046551.2
*IL1B*	Interleukin 1 beta	F: CTGAGGAGCATCCTTTCATTC R: GTCCTGGAGTTTGCACTTTAT	114	97	NM_174093.1
*IL6*	Interleukin 6	F: AGTGTGAAAGCAGCAAGGAGA R: AGCAAATCGCCTGATTGAAC	105	112	NM_173923.2
*LTA4H*	Leukotriene A4 hydrolase	F: GTCAGTGCCAGGCTATCCAC R: TCTTTGGGGACAGACACCTC	97	92	NM_001034280.1
*PTGES3*	Prostaglandin E synthase 3	F: TTGAGGAAAGCGAGAAGAGG R: AAGCAGGTTGCATCGTGAA	146	93	NM_001007806.2
*PTGS2*	Prostaglandin-endoperoxidase synthase-2	F: GGTGTGAAAGGGAGGAAAGA R: GGCAAAGAATGCAAACATCA	117	93	NM_174445.2
*TNF*	Tumor necrosis factor alpha	F: TGTAGCCGACATCAACTCTCC R: CCCTGAAGAGGACCTGTGAG	149	103	NM_173966.3

^1^F = Forward, R = Reverse

^2^Efficiency = − 1 + 10^(−1/slope)^ × 100

^3^National Center for Biotechnology Information (NCBI; https://www.ncbi.nlm.nih.gov/)

### Statistical analysis

All statistical analyses were performed in R v.4.2.2 [23] using the lme4 package [24] for linear mixed models and the emmeans package [25] to generate pairwise contrasts. A Sidak’s adjustment was used to correct for multiple comparisons. For viability, data were analyzed within each time. For both viability and real time data, CBD exposure treatment was considered the fixed effect and animal was considered random. The ΔCt of target gene expression was used for statistical analysis. Statistical significance was declared at P < 0.05, with P < 0.1 considered a tendency.

## Results

### Viability

**Fig. 3:**
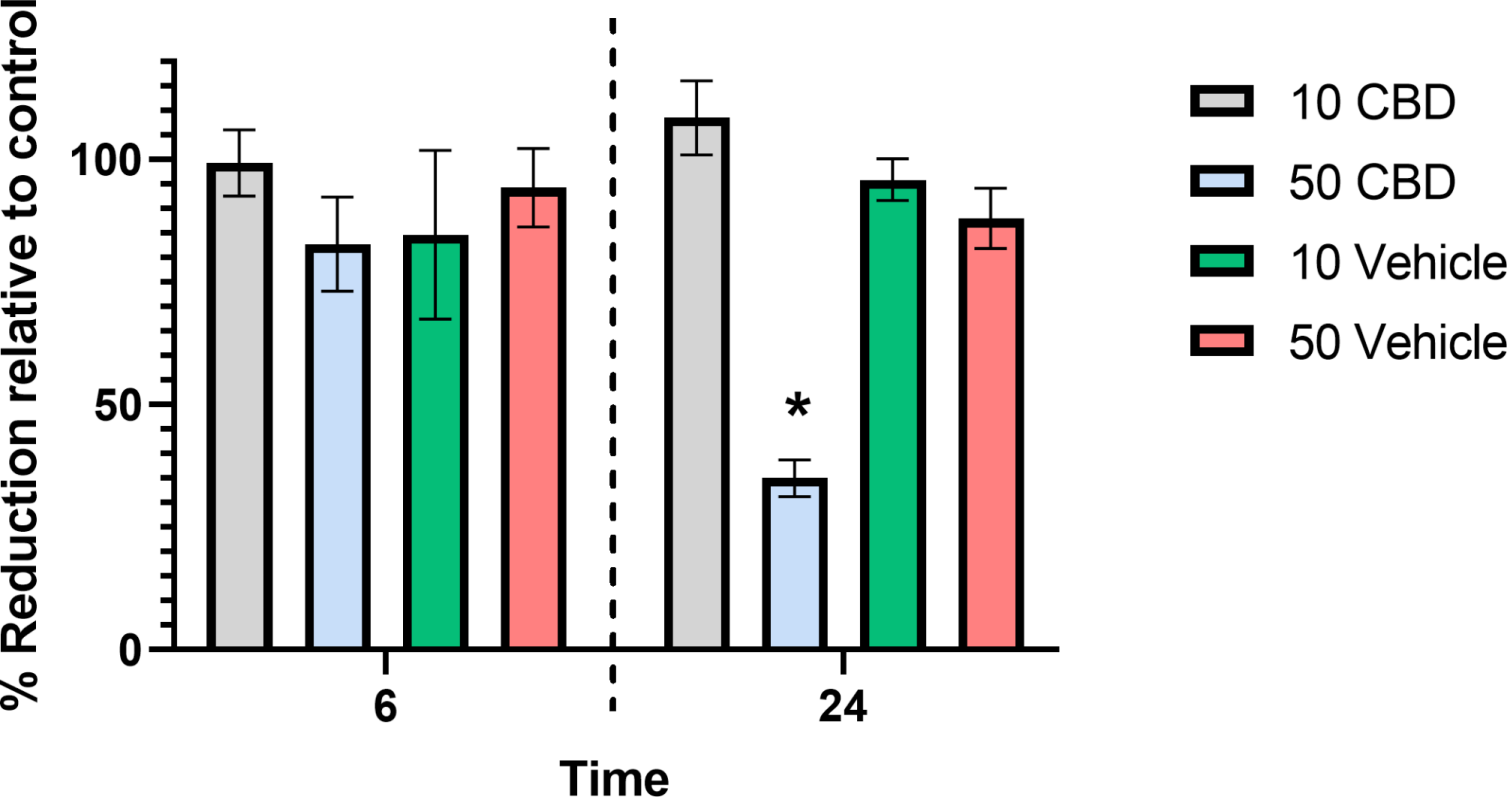
Viability of ruminal epithelial cells as indicated by the percent reduction in alamarBlue relative to control, for cells exposed to 10 μM, 50 μM cannabidiol (CBD) or equivalent levels of vehicle only (1:1 methanol:DMSO), for either 6 or 24 h. Asterisk indicated significance (P < 0.05)

Cannabidiol did not affect viability of REC at a concentration of 10 μM, for either 6 or 24 h (Fig. 3). While 50 μM CBD had no detrimental effect after 6 h exposure, there was approximately a 35% reduction of resazurin to resorufin at 24 h relative to the corresponding media only control, equating to a 65% inhibition of cell growth. Equal amounts of vehicle in the absence of CBD did not affect cell viability at either time point assessed.

### Experiment 1: CBD as a treatment for existing inflammation

**Fig. 4:**
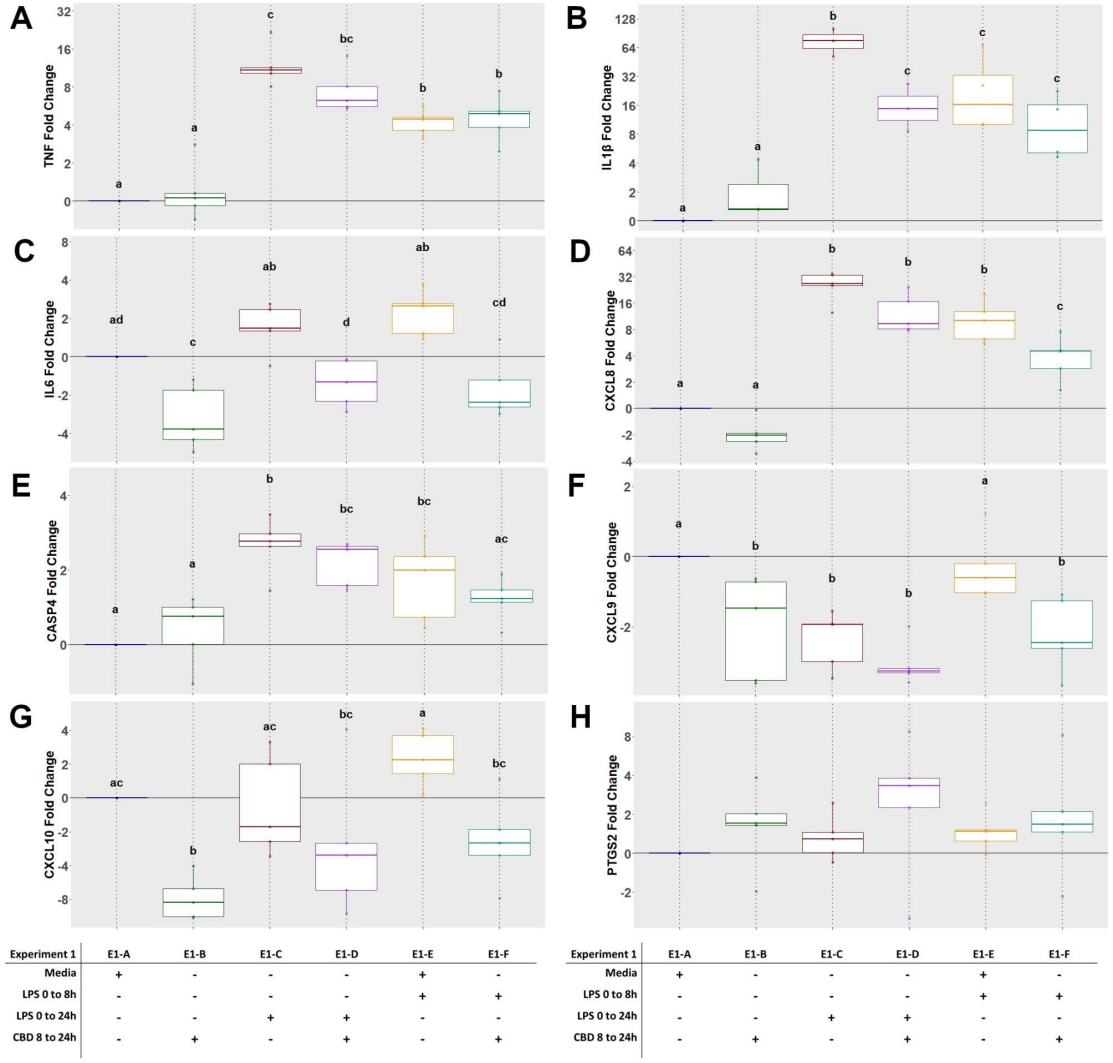
Experiment 1 gene expression of pro-inflammatory cytokines *TNF* (**A**), *IL1β* (**B**), *IL6* (**C**) and *CXCL8* (**D**), inflammasome regulator *CASP4* (**E**), chemokines *CXCL9* (**F**) and *CXCL10* (**G**), and prostaglandin synthase *PTGS2* (**H**) in primary ruminal epithelial cells in various exposure combinations of cannabidiol (CBD) and lipopolysaccharide (LPS). E1-A through E1-F indicate treatment groups. The ΔCt was used for statistical analysis and results are presented as fold change with exposure treatments held relative to CON within animal. Different letters indicate significance (*P* < 0.05)

Exposure to LPS (E1-C) for 24 h resulted in upregulation of *TNF*, *IL1B*, *CXCL8* and *CASP4* by 13-, 93-, 29- and 4-fold, respectively (P < 0.05) relative to untreated control (Fig. 4A, B, D and E). When LPS was added to the cell media and then removed after 8 h (E1-E group), expression of *TNF*, *IL1B* and *CXCL8* remained greater, at 5-, 29-, and 14-fold, respectively, relative to control, but was not the same magnitude as 24 h of LPS exposure (Fig. 4A, B and D). The addition of CBD following 8 h of LPS exposure (E1-D) resulted an upregulation of *IL1B*, but the level of expression was significantly less than LPS (E1-C) alone (27- vs. 93-fold; P = 0.002). However, expression of *TNF*, *CXCL8*, *CASP4*, *CXCL9* and *CXCL10* for the E1-C and E1-D groups was similar (Fig. 4A, D, E, F and G). Following removal of LPS after 8 h and the addition of CBD for 16 h (E1-F), the expression level relative to control of *CXCL8* was less than E1-E (5- vs. 14-fold; P = 0.011), but this effect was not observed for *TNF*, *IL1B* or *CASP4*. *CXCL9* was down-regulated (1.8-fold; P = 0.005 for the E1-F group compared to control, and expression of both *CXCL9* and *CXCL10* was also less for E1-F compared to E1-E (P < 0.05). Expression of *TNF*, *IL1B* and *CXCL8* was not affected by exposure to CBD alone (E1-B). Expression of *IL6* was not influenced by exposure to LPS, however there was a 3-fold down-regulation observed with the addition of CBD relative to control (Fig. 4C; P < 0.001). Similarly, both *CXCL9* and *CXCL10* were down-regulated 1.5- and 2.9-fold, respectively with CBD exposure compared to CON. Additionally, *CXCL9* was downregulated for E1-C, E1-D and E1-F (P < 0.05), but E1-E was not different then control. There were no effects of treatments on expression levels of *PTGS2* (Fig. 4H), *PTGES3* or *LTA4H* (Data not shown for *PTGES3* and *LTA4H*).

### Experiment 2: CBD as a prophylactic measure against inflammation

**Fig. 5:**
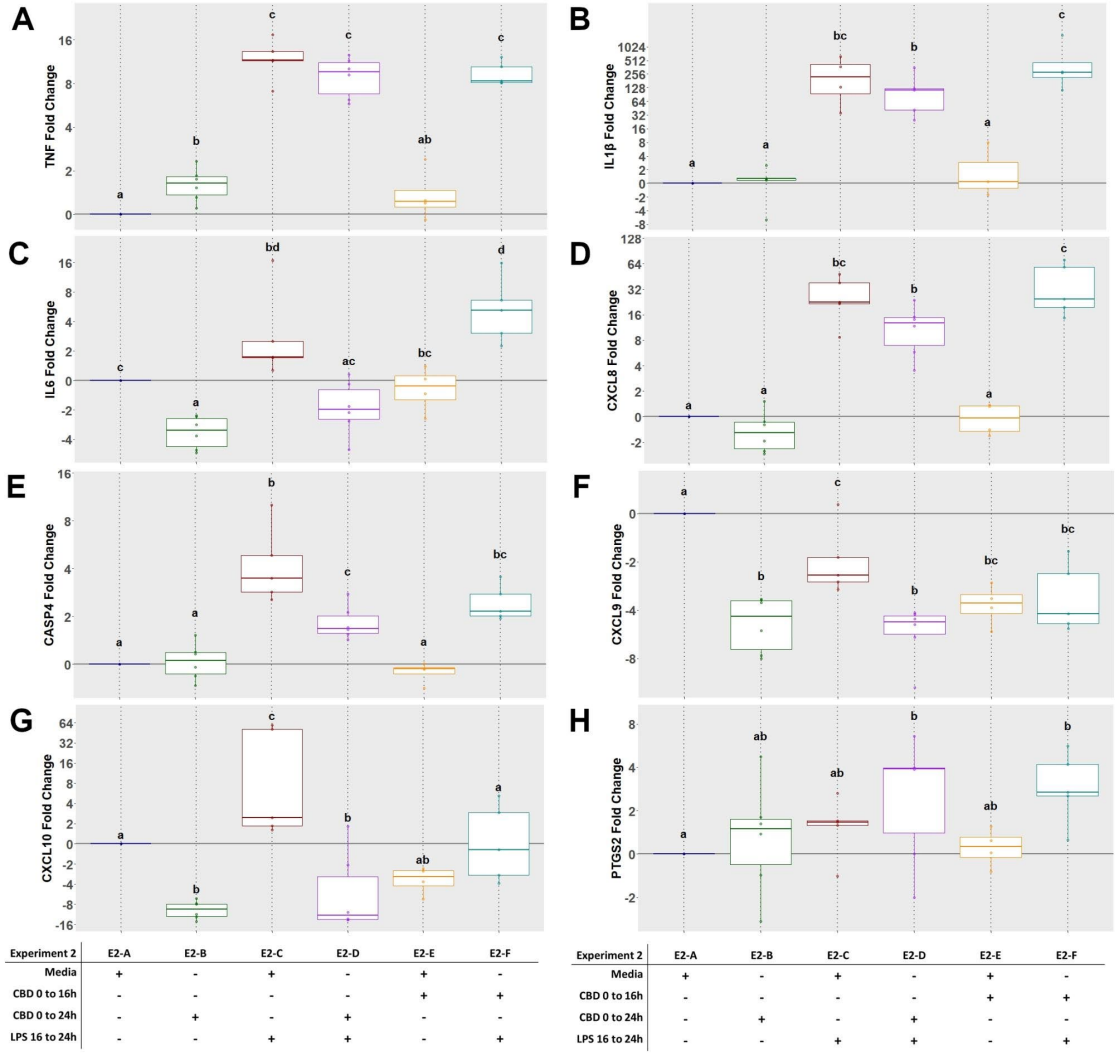
Experiment 2 gene expression of pro-inflammatory cytokines *TNF* (**A**), *IL1β* (**B**), *IL6* (**C**) and *CXCL8* (**D**), inflammasome regulator *CASP4* (**E**), chemokines *CXCL9* (**F**) and *CXCL10* (**G**), and prostaglandin synthase *PTGS2* (**H**) in primary ruminal epithelial cells in various exposure combinations of cannabidiol (CBD) and lipopolysaccharide (LPS). E1-A through E1-F indicate treatment groups. The ΔCt was used for statistical analysis and results are presented as fold change with exposure treatments held relative to CON within animal. Different letters indicate significance (*P* < 0.05)

Compared to control, expression of *TNF*, *IL1B*, *IL6*, *CXCL8*, *CASP4* and *CXCL10* was upregulated (16-, 240-, 25-, 6-, 33- and 8-fold, respectively; P < 0.05) following exposure to LPS (Fig. 5A, B, C, D, E and G). When CBD was added for 16 h prior to LPS exposure (E2-D), expression of *IL6*, *CXCL10* and *CASP4* was less compared to LPS alone (E2-C; P < 0.05), however this effect was not detected for *TNF*, *IL1B*, *CXCL8* or *CXCL9*. Additionally, CBD alone (E2-B) resulted in a 2- and 6-fold decrease in expression of *IL6* (P = 0.003) and *CXCL10* (P = 0.002), respectively, compared to control. Expression of *CXCL9* (Fig. 5F) was down-regulated 2-fold when exposed to LPS alone compared to control (P = 0.012), which was greater than the E2-B and E2-D groups (P < 0.05). Compared to the E2-D treatment, E2-F had a greater magnitude of expression change for *IL1B* (104- vs. 544-fold; P = 0.001) and *CXCL8* (12- vs. 38-fold; P = 0.006). This same effect was also observed for *IL6*, where there was a 1.3-fold down-regulation for E2-D and a 9-fold upregulation for E2-E (P < 0.001). Additionally, there was a 5-fold increase in expression of *PTGS2* for both E2-D and E2-F treatment groups (Fig. 5H; P < 0.05). Expression of *PTGES3* and *LTA4H* was not affected by treatments (Data not shown).

## Discussion

Cannabidiol is an abundant phytocannabinoid and one of dozens of similar terpenophenolic compounds produced by hemp plants [26]. Phytocannabinoids interact, in different capacities, with the endocannabinoid system, which includes the G protein-coupled receptors CB1 and CB2, and several endogenous ligands, which are enzymatically mediated, primary through fatty acid amide hydrolase and monoacylglycerol lipase [7, 27]. In recent years, there has been increased interest in CBD for its possible health benefits. The putative bioactivities of CBD in humans and rodents are numerous, and include potential immuno-modulatory effects in intestinal epithelial cells [14]. Of particular interest for livestock species is the potential for CBD to mitigate detrimental effects of inflammation in the gastrointestinal epithelium [14]. However, the effects of CBD in the ruminant gastrointestinal tract have not been evaluated. In the present experiment, we employed a cell culture model using bovine, primary REC to first determine if there is a cytotoxic effect of CBD, and to investigate both the potential therapeutic and prophylactic effects of CBD under LPS-induced inflammatory conditions.

While CBD has been utilized in previous cell culture models [28, 29], a recent review of the literature [30] suggests that viability of cells may be affected in a dose-dependent manner when exposed to CBD. The degree of cytotoxicity also appears to be influenced by the cell type. As such, we evaluated the effect of two different levels of CBD, for varying lengths of time, on REC viability using an alamarBlue assay. This assay quantifies the cellular reduction of resazurin to resorufin, and is therefore reflective of cell metabolic activity, and does not directly measure cell death or apoptosis. Nonetheless, it is routinely used as an estimate of cell viability [31], and the data in the present study are interpreted as such. While the lower dose of CBD (10 μM) did not affect cell viability for either of the two time points, there was a significant cytotoxic effect when cells were exposed to 50 μM for 24 h. This effect was not observed when the cells were incubated with the vehicle alone, suggesting that the CBD was solely responsible for the reduction in viability. These results are consistent with Olah et al. [29], where a CBD concentration of 50 μM resulted in apoptosis-driven cytotoxicity in human sebocytes. While negative effects of high doses of CBD have been reported in several studies [32, 33], other studies indicate that 10 μM or less does not affect cell viability [30].

As previously demonstrated by Kent-Dennis et al. [20], when exposed to LPS in culture, there is substantial upregulation of pro-inflammatory gene expression REC. In both experiments conducted, pro-inflammatory cytokines *TNF*, *IL1B*, and *CXCL8* were upregulated in response to LPS alone. In Experiment 1, when LPS was removed from the media after 8 h, all three of these genes returned to baseline by 24 h. This is consistent with effects observed by Kent-Dennis et al. [20], where pro-inflammatory cytokines were no longer upregulated when LPS was removed after 12 h of exposure followed by a 36-h recovery period. In Experiment 2, *IL6* and *CXCL10* were upregulated in response to 8 h of LPS, whereas this effect was not observed in Experiment 1, where LPS exposure was 24 h in duration, suggesting tighter regulation of these genes. Overall, the cytokine expression data confirm the induction of an inflammatory response.

The effects of CBD, independent of LPS, on REC were evaluated, with cells exposed for 16 h in Experiment 1 and 24 h in Experiment 2. Consistent between both experiments, CBD resulted in a down-regulation of *IL6*, *CXCL9* and *CXCL10* relative to resting state, indicating immunomodulation, even in the absence of an inflammatory stimuli. Expression of *IL6*, a cytokine that is associated with the onset of an inflammatory event [34], is consistently found to be suppressed by CBD [35, 7]. Down-regulation of the interferon-induced chemokines, *CXCL9* and *CXCL10*, may indicate the involvement of cannabinoids in immune tolerance via interaction with the receptor CXCR3, which has previously been observed in mouse small intestinal mucosa [36]. Activation of the endocannabinoid system, either through endogenous or phytocannabinoids, is associated with the regulation of immune responses, and it is often considered a “gate-keeping” mechanism [37], however exact mechanisms by which CBD regulates transcription and production of pro-inflammatory molecules have not been fully elucidated. Previous work has implicated a number of different transcription factors in modulating this response. For example, PPARs, which are mediated by the endocannabinoids system [38], have been shown to suppress *IL6* transcription [34]. Kozela et al. [35] suggested that CBD could act by interfering with the JAK-STAT pathway or by altering phosphorylation of IkB in the TLR4 pathway, thereby preventing NF-kB translocation. Although these mechanisms have been discussed solely in the context of a pro-inflammatory response, the results of the current study nonetheless indicate immune activation and suggest a potential prophylactic effect of CBD.

A recent systematic review, analyzing the results from studies investigating the effects of cannabinoids on inflammation in laboratory animals, reported consistent reductions in pro-inflammatory cytokines when CBD was administered [39]. Recent reports in various tissue types have similarly shown suppression of inflammatory mediators [40, 41] Using a pro-inflammatory model in colonic explants, Couch et al. [14] demonstrated that CBD was able to ameliorate the production of inflammatory cytokines, both when CBD was added to the media at the same time as IFNy and *TNF*a (added to induce inflammatory response) as well as when added to a previously inflamed tissue. To assess the therapeutic benefits in ruminal epithelial cells, we utilized a culture model with LPS stimulation followed by treatment of CBD. In this context, CBD reduced expression of *IL1B*, suggesting that a post-stimulation treatment can suppress the pro-inflammatory response. Previous work has provided evidence for *IL1B* regulation by cannabinoids [42]. Specifically, CBD has been shown to inhibit NLRP3-mediated *IL1B* activation [43]. To further explore the effects of *IL1B* expression by CBD, expression of *CASP4* was analyzed in the present study. *CASP4*, a protease that regulates pyroptosis, controls CASP1 and inflammasome activation [44] and therefore is involved in the production of *IL1B* [45]. However, in the present experiment, while *CASP4* expression was upregulated by LPS, it was not affected by CBD exposure. It is possible, therefore, that CBD acts on CASP1 directly [46]. Surprisingly, *TNF* and *CXCL8* were not affected by the addition of CBD. Consistent with our results, CBD did not suppress production of cytokines in Caco2 cells under inflammatory conditions [14]. Henshaw et al. [39] also found inconsistencies in the production of pro-inflammatory cytokines when CBD was administered.

Lipopolysaccharide-derived inflammatory response also activates the JAK-STAT pathway by way of interferon induction, which leads to transcription of specific chemokines, including *CXCL9* and *CXCL10* [47]. This pathway has also been proposed as a target for CBD bioactivity [48]. In the present experiment, expression of *CXCL10* was not affected following prior exposure of REC to LPS. Cannabidiol in the media, alone or in combination with LPS, resulted in a down-regulation of *CXCL9*. However, there was also a down-regulation with LPS alone. The reason for this response in unknown, however it may represent a regulatory mechanism, in order to prevent a prolonged inflammatory activity, often observed in cells subjected to repeated or prolonged bouts of LPS exposure [20, 49].

Removal of LPS after 8 h, with only media added for the last 16 h, resulted in a greater capacity to reduce *CXCL8*, suggesting that when the inflammatory stimulus was removed, there was rapid remediation compared to when CBD was added. *CXCL8* is a tightly regulated and potent neutrophil chemoattractant [50]. These results may indicate that although *CXCL8* was not suppressed when LPS and CBD were added together, once the stimulus is removed, CBD curtails the acute inflammatory response. This suggests a possible regulatory mechanism of CBD that aids in preventing prolonged inflammation which may cause damage to the cells. Indeed, *CXCL8* has long been a target for developing therapies for inflammatory disorders [51], including chronic diseases of the gastrointestinal tract [52], and CBD has been recognized for its potential in regulating *CXCL8* activities [53, 54]. Together these results suggest that CBD may be a promising target to investigate as a therapeutic supplement for mitigating digestive disorders such as ruminal acidosis.

Given the apparent immunomodulatory effects of CBD on an established immune response, we subsequently investigated its prophylactic capacity. In Experiment 2, cannabidiol was therefore added to the media for 16 h prior to exposing the cells to LPS. Of the cytokines, *IL6* was suppressed when CBD was added as a preventative measure, with its expression being significantly less compared to LPS alone. Additionally, both *CASP4* and *CXCL10* were expressed at lower levels when CBD was added prior to LPS compared to LPS alone. These data suggest a preventative effect of CBD. This is consistent with Olah et al. [29] showing that CBD prevented the inflammatory response related to acne development in human sebocytes. Moreover, in a model of colitis in mice, a synthetic cannabinoid, AM841, was effective at both a preventative and a treatment for colitis-related inflammation [13]. In ruminant livestock, especially those that experience stresses such as those occurring during transport or co-mingling in a feedlot, a compound that could be used as a preventative to reduce the severity of inflammation could be valuable.

Expression of *CXCL9* was again suppressed by LPS in Experiment 2, although not to the same degree as when REC were exposed to CBD. As the cells were only exposed to LPS for 8 h in Experiment 2, versus 24 h in Experiment 1, this may further suggest a regulatory mechanism for chemokine activity. When CBD was removed prior to the addition of LPS, the subsequent increase in *IL1B*, *CXCL8*, *IL6* and *CXCL10* was greater than cells incubated with CBD before and during LPS exposure, suggesting that inflammatory suppression is CBD-dependent and wanes quickly following its removal. Together these results suggest that CBD may possess the ability to suppress an inflammatory response, however the response appears to be temporally specific. That is, the response may depend on on the timing, as well as the duration, of the dose in relation to the inititation of inflammation.

Previous work has shown that *PTGS2* expression increases in response to LPS exposure in REC [20]. *PTGS2* is a cyclooxygenase that mediates synthesis of prostaglandins during the inflammatory response [52]. However, in the present study, *PTGS2* was not affected by LPS alone for either experiment. The reason for this discrepancy is unknown, however, as these experiments were conducted with primary cells, increased between-animal variation is expected and may lead to inconsistencies. Additionally, the animals used in the present study were more mature compared previous work [20], and either age or prior inflammatory events may dampen this response. While the effects were only numerical in the first experiment, *PTGS2* was upregulated when REC were exposed to both LPS and CBD in experiment 2. Eicosanoids are intertwined with the ECS, with endocannabinoids being substrates for prostaglandin synthesis [56]. The immunomodulatory effects of phytocannabinoids on the arachidonic acid-associated pathways (mainly prostaglandin and leukotriene pathways) are varied, and are dependent on type of compound, dose, cell type and physiological status of the cells or tissue [57, 58]. In lung fibroblasts, administration of cannabinoids, including THC, CBN and CBD, into the cell media resulted in stimulation of PGE2 synthesis [59]. Other studies have also shown inhibitory effects of cannabinoids on *PTGS2* expression under inflammatory conditions [60]. To further explore the downstream effects of CBD on the arachidonic acid pathway in REC, expression of *PTGES3* and *LTA4H*, key enzymes in the synthesis of specific prostaglandins and leukotrienes, respectively, were evaluated. However, these two genes were not affected by any of the treatments. Precise mechanisms for cross-talk between the eicosanoid and endocannabinoid systems are complex [55]. A more in depth analysis is required to fully elucidate the role of CBD in prostaglandin synthesis in REC. In particular, it would pertinent to understand the interactions between cannabinoids and specific prostaglandins.

## Conclusions

In conclusion, the present study shows that CBD exerts direct bioactive effects on REC by influencing the inflammatory response to LPS. Specifically, as a potential treatment for an established immune response, CBD reduced the magnitude of IL1B expression following exposure to LPS, indicating a capacity for CBD to mitigate a pro-inflammatory response. Moreover, a preventative effect was observed when REC were treated with CBD prior to the LPS insult, as demonstrated by lower expression of *IL6*, *CASP4* and *CXCL10*, however the effect was dependent on the presence of CBD. In the REC, a lower dose of 10 μM CBD was effective at eliciting a response without negatively affectively viability. However, there was a cytotoxic effect when cells were exposed to 50 μM for 24 h. A limitation of the current study is the possibility that the in vitro results do not directly translate to multicellular tissues or whole, live animals. Future experiments with animals must therefore establish an effective in vivo dose of CBD, to evaluate any potential negative effects on the gastrointestinal epithelial tissue. Another constraint of the present study is that the REC were not grown as polarized monolayers, which may have provided additional insight into physiological effects of CBD on inflammation. Overall, this study provides evidence that CBD may be useful both as a treatment for established immune response and as a prophylactic, which would help to mitigate or prevent the negative consequences of inflammation. The results provide justification for further exploration into the potential benefits of cannabinoids in ruminants. Specifically, further research should aim to investigate the possible use of phytocannabinoids as feed additives or supplements, in order to improve health and productivity of the animals.

## Data Availability

The data supporting this study’s findings are available from the corresponding author upon reasonable request.

## List of abbreviations

CBD: Cannabidiol
DPBS: Dulbecco’s phosphate buffered saline
LPS: Lipopolysaccharide
MEM: Minimum essential media
REC: Ruminal epithelial cells
THC: Delta-9- tetrahydrocannabinol
